# Global burden of myocarditis and cardiomyopathy in children and prediction for 2035 based on the global burden of disease study 2019

**DOI:** 10.3389/fcvm.2023.1173015

**Published:** 2023-05-02

**Authors:** Hongjun Ba, Daoqi Zhang, Shiyang Guan, Jinxin Zheng

**Affiliations:** ^1^ Department of Pediatric Cardiology, Heart Centre, The First Affiliated Hospital, Sun Yat-sen University, Guangzhou, China; ^2^ Key Laboratory on Assisted Circulation, Ministry of Health, Guangzhou, China; ^3^ Department of Internal Medicine Teaching and Research Section, Xuancheng Vocational and Technical College, Xuanchen, China; ^4^ Second Affiliated Hospital of Anhui Medical University, Hefei, China; ^5^ Department of Nephrology, Ruijin Hospital, Shanghai Jiao Tong University School of Medicine, Shanghai, China; ^6^ Chinese Center for Disease Control and Prevention (Chinese Center for Tropical Diseases Research), NHC Key Laboratory of Parasite and Vector Biology, WHO Collaborating Centre for Tropical Diseases, National Center for International Research on Tropical Diseases, National Institute of Parasitic Diseases, Shanghai, China

**Keywords:** sociodemographic index (SDI), childhood, myocarditis, cardiomyopathy, global burden of disease, prediction

## Abstract

**Background:**

Myocarditis and cardiomyopathy are commonly occurring cardiovascular diseases that seriously threaten children's health. It was urgent to update the global incidence and mortality of childhood myocarditis and cardiomyopathy, and to predict the incidence rate of 2035 by the Global Burden of Disease database.

**Methods:**

The Global Burden of Disease study data from 1990 to 2019 in 204 countries and territories were used to determine: global incidence and mortality rates of childhood myocarditis and cardiomyopathy from 0 to 19 by five age groups; relationship between sociodemographic index (SDI) and incidence and mortality rates by age group; and, based on an age-period-cohort model, the projected incidence of childhood myocarditis and cardiomyopathy for 2035.

**Results:**

From 1990 to 2019, global age-standardized incidence rate decreased by 0.1% (95% UI 0.0–0.1) to 7.7% (95% UI 5.1–11.1). Boys had higher age-standardized incidence of childhood myocarditis and cardiomyopathy than girls [9.12, (95% UI 6.05–13.07) vs. 6.18, (95% UI 4.06–8.92)]. Childhood myocarditis and cardiomyopathy affected 121,259 (95% UI 80,467–173,790) boys and 77,216 (95% UI 50,684–111,535) girls in 2019. At the regional level, SDI changes in most areas showed no meaningful difference. In East Asia and high-income Asia Pacific, increased SDI was associated with decreased and increased incidence rate, respectively. In 2019, 11,755 (95% UI 9,611–14,509) children died from myocarditis and cardiomyopathy worldwide. Age-standardized mortality rate decreased significantly by 0.4% (95% UI 0.2–0.6)–0.5% (95% UI 0.4–0.6). Number of deaths from childhood myocarditis and cardiomyopathy in 2019 was highest in the <5-year-old group [7,442 (95% UI 5,834–9,699)]. Myocarditis and cardiomyopathy incidence in 10–14- and 15–19-year-olds is projected to increase by 2035.

**Conclusion:**

Global data on childhood myocarditis and cardiomyopathy from 1990 to 2019 showed a decreasing trend in incidence and mortality, and an increasing trend in older children, especially in high SDI regions.

## Introduction

Cardiovascular diseases, the single largest cause of mortality and morbidity globally, constitute a significant burden in terms of disability and lost adult productivity (1). The incidence and mortality due to myocarditis and cardiomyopathy are much higher in children (2). Myocarditis and cardiomyopathy are a serious threat to the health of children and adolescents and a huge burden on society. Myocarditis represents an often-underdiagnosed cardiovascular disease that causes several life-threatening conditions, including acute heart failure and dilated cardiomyopathy, and even sudden death (3, 4). Incidence and specific causes of myocarditis vary widely worldwide (5, 6) and in developed countries, myocarditis is usually caused by viral pathogens. In developing countries and environments with limited resources, the causes of myocarditis are dominated by rheumatic heart disease and infectious agents (such as *Trypanosoma cruzi)* and diphtheria (7). Cardiomyopathy is associated with nearly 50% sudden deaths in heart transplant-listed patients during childhood or adolescence (8).

Globally, childhood myocarditis and cardiomyopathy clinical presentations and complications include severe cardiac failure, arrhythmias, and growth retardation (2, 8). The burden of childhood myocarditis and cardiomyopathy is substantial in high-income countries. Overall, medical expenditure due to severe myocarditis and cardiomyopathy is high (9). Establishing a model of health care management that will reduce the medical costs of childhood myocarditis and cardiomyopathy is recommended (10, 11). Children with myocarditis and cardiomyopathy need intensive monitoring and disease management to reduce the number of deaths and to control the symptoms (12). Improving the quality of medical care requires substantial knowledge about the current burden of the disease and its future trend. The Global Burden of Disease (GBD) study dataset is useful for analysing disease characteristics, because the dataset includes reliable data on childhood myocarditis and cardiomyopathy from 1990 to 2019. The findings of such data analyses can help to inform regional and national health policies (11). To date, there are no studies on the burden of childhood myocarditis and cardiomyopathy.

In this study, the incidence and mortality rates of childhood myocarditis and cardiomyopathy using the GBD data were estimated from 1990 to 2019; the data were stratified by age, sex, sociodemographic index (SDI), region and country. A global prediction was made by the expected incidence rate for 2035 based on the GBD study 2019.

## Materials and Methods

### Data sources

We conducted a secondary analysis of GBD 2019 data, which used all available up-to-date epidemiological data and standardized methods to compare health losses from 359 diseases and injuries by age group and sex in 204 countries and territories. Patients or the public were not involved in the design, or conduct, or reporting, or dissemination plans of our research. GBD uses a variety of interrelated indicators to measure population health losses, including death and incidence rates. For this report, we extracted the incidence and death rates and their 95% uncertainty intervals (UI) as measures of childhood myocarditis and cardiomyopathy burden, from GBD 2019 using the GBD results tool. The data included locations, age groups, sex, and death and incidence numbers, we stratify GBD data by age, sex, sociodemographic index (SDI), region and country. Myocarditis and cardiomyopathy occurrences were identified using the International Classification of Diseases version 10 (ICD-10) codes. Disease coded as B33.2–B33.20, B33.22–B33.24, D86.85, I40–I41.8, I42–I43.8, and I51.4–I51.6 was identified as myocarditis and cardiomyopathy (https://ghdx.healthdata.org/record/ihme-data/gbd-2019-cause-icd-code-mappings).

Detailed information on childhood myocarditis and cardiomyopathy can be found at http://ghdx.healthdata.org/gbd-results-tool. The SDI is a summary measure that reflects the sociodemographic development, including local income, average educational attainment, and total fertility rates (13). SDI values range from 0 (lowest income, lowest educational attainment, and highest fertility rate) to 1 (highest income, highest educational attainment, and lowest fertility rate) (14–16). The 204 countries and territories in the GBD study were classified into high-, high-middle-, middle-, low-middle-, and low-SDI regions. The cut-off values that were used to determine quintiles for the analysis were computed using the 2019 country-level SDI estimates.

The childhood age group in this study encompasses children and adolescents aged 0–19 years (17). Data of children with myocarditis and cardiomyopathy aged 0–19 years from 1990 to 2019 were extracted from the GBD database. These patients' ages were divided into four age subgroups (under 5, 5–9, 10–14, and 15–19 years).

### Statistical analysis

The standardized methods of GBD 2019 were published by the GBD team and extensively reported elsewhere (14, 18). Incidence and mortality rates for childhood myocarditis and cardiomyopathy for 204 countries and territories from 1990 to 2019 were estimated by age and sex using a Bayesian meta-regression model in DisMod-MR 2.1 (Dr. Jan J Barendregt, EpiGear). During data processing, the mean of 1,000 draws was generated for all reported data, and the 2.5th and 97.5th percentiles of the ordered draw represent the 95% UIs.

Associations of age-standardized incidence with the SDI for the 204 countries and territories and 21 GBD regions were evaluated by smoothing spline models (19). Age-period-cohort models were performed to predict myocarditis and cardiomyopathy incidence and case numbers through to 2035. The prediction was conducted in R software V.4.0.2 (R Core Team, Vienna, Austria) using the NORDPRED package (Version 0.0.36) (20), which performs well for projecting the current trends in myocarditis and cardiomyopathy incidence to the future. The number of new cases predicted for the year 2035 were determined by taking a weighted average of the projected incidence rates for the last two prediction periods, centering on 2035, and then applying the rates to the available United Nations population forecasts for each country for that year. All statistical analyses were performed in R software to estimate the incidence rates and numbers, using the GBD dataset.

## RESULTS

### Global burden

**Figure 1:**
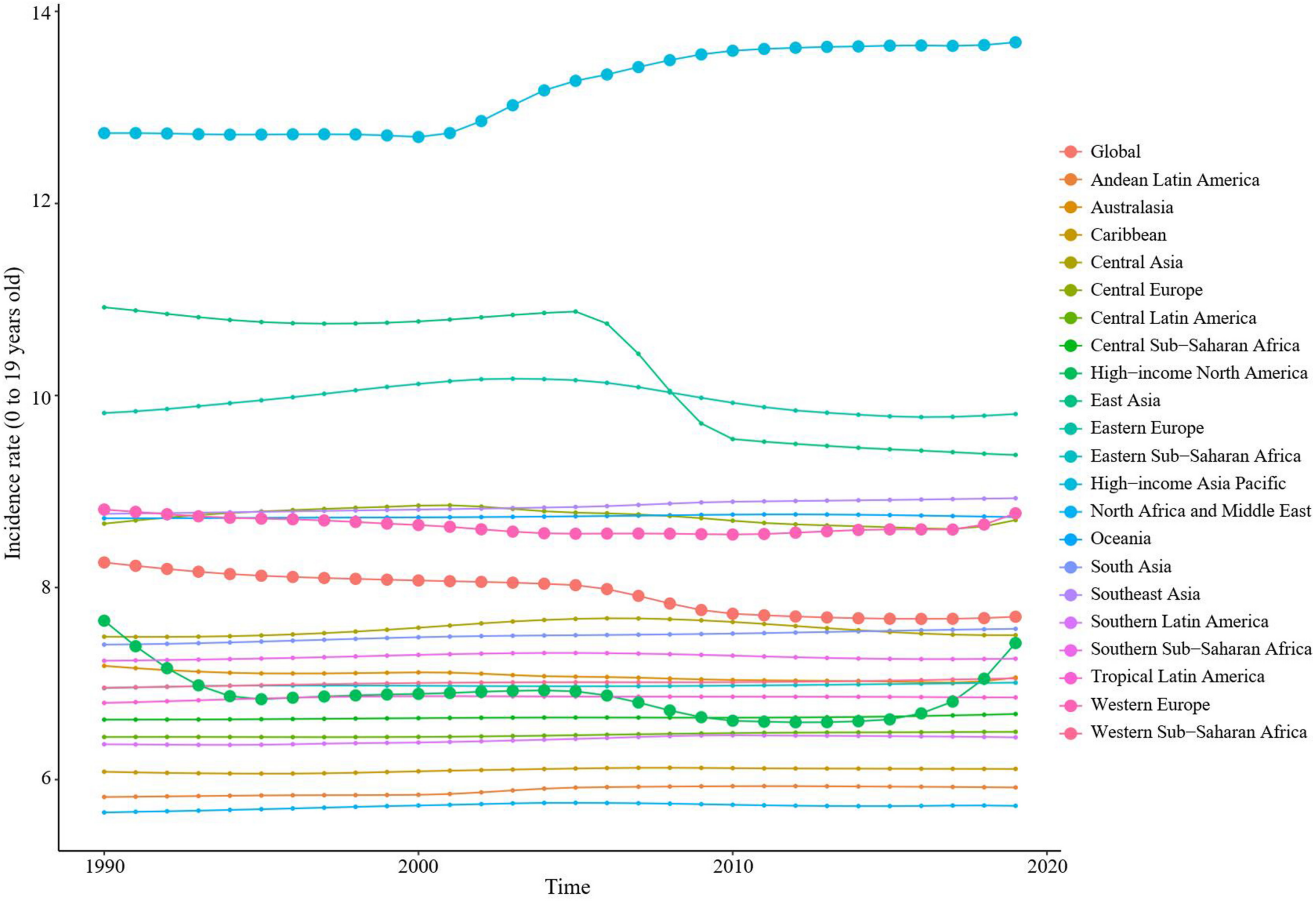
Trends in incidence of childhood myocarditis and cardiomyopathy from 1990 to 2019 in 21 regions worldwide. From 1990 to 2019, the incidence of childhood myocarditis and cardiomyopathy declined globally. The incidence of childhood myocarditis and cardiomyopathy did not change significantly in most regions, but increased in high-income Asia Pacific, high-income North America and Western Europe.

From 1990 to 2019, the global age-standardized incidence rate of childhood myocarditis and cardiomyopathy decreased by 0.1% (95% UI 0.0–0.1) to 7.7% (95% UI 5.1–11.1) (Table 1). From 1990 to 2019, the incidence of childhood myocarditis and cardiomyopathy declined globally and did not change significantly in most regions, but increased in high-income Asia Pacific, high-income North America and Western Europe (Figure 1 and Supplementary Table S1). Globally, in 2019, the age-standardized incidence of childhood myocarditis and cardiomyopathy was higher in boys than in girls. Childhood myocarditis and cardiomyopathy affected 121,259 (95% UI 80,467–173,790) boys and 77,216 (95% UI 50,684–111,535) girls in 2019 (Table 1).

**Table 1:** Percent change in age-standardized rates of childhood myocarditis and cardiomyopathy by age group, sex and SDI, 1990–2019.

	Deaths (95% UI)		Incidence (95% UI)	
	2019 age standardized rates (per 100,000)	Percent change in age standardized rates, 1990–2019	2019 age standardized rates (per 100,000)	Percent change in age standardized rates, 1990–2019
**Age (years)**				
Under 5	1.12 (0.88–1.46)	−0.5(−0.69–−0.17)	6.88 (4.3–9.77)	−0.07(−0.08 to −0.06)
5–9	0.18 (0.15–0.22)	−0.28 (0.52 to −0.02)	6.97 (3.71–11.02)	−0.08(−0.09 to −0.07)
10–14	0.19 (0.16–0.23)	−0.24(−0.45–0.04)	7.76 (4.53–12.29)	−0.07(−0.09 to −0.06)
15–19	0.32 (0.26–0.38)	−0.21(−0.38 to −0.07)	9.27 (5.30–14.92)	−0.07(−0.09 to −0.04)
**Sex**				
Male	0.51 (0.40–0.66)	−0.42(−0.10 to −0.64)	9.12 (6.05–13.0)	−0.07(−0.09 to −0.06)
Female	0.40 (0.32–0.49)	−0.50(−0.25 to −0.67)	6.18 (4.06–8.92)	−0.07(−0.08 to −0.05)
**Sociodemographic factor**				
Global	0.50 (0.40–0.60)	−0.40(−0.60 to −0.20)	7.70 (5.10–11.10)	−0.10(−0.10–0)
High SDI	0.30 (0.30–0.40)	−0.50(−0.60 to −0.50)	8.80 (5.90–12.30)	0(−0.10–0)
High-middleSDI	0.40 (0.30–0.50)	−0.50(−0.70 to −0.30)	8.30 (5.50–11.80)	−0.10(−0.10–0)
Middle SDI	0.40 (0.40–0.60)	−0.50(−0.70 to −0.20)	7.90 (5.20–11.40)	−0.10(−0.10 to −0.10)
Low-middleSDI	0.30 (0.20–0.40)	−0.40(−0.60 to −0.10)	7.40 (4.90–10.80)	0(−0.10–0)
Low SDI	0.70 (0.50–1.0)	−0.40(−0.60–0)	7.00 (4.60–10.10)	−0.10(−0.10–0)

UI, uncertainty interval; SDI, sociodemographic index.

**Figure 2:**
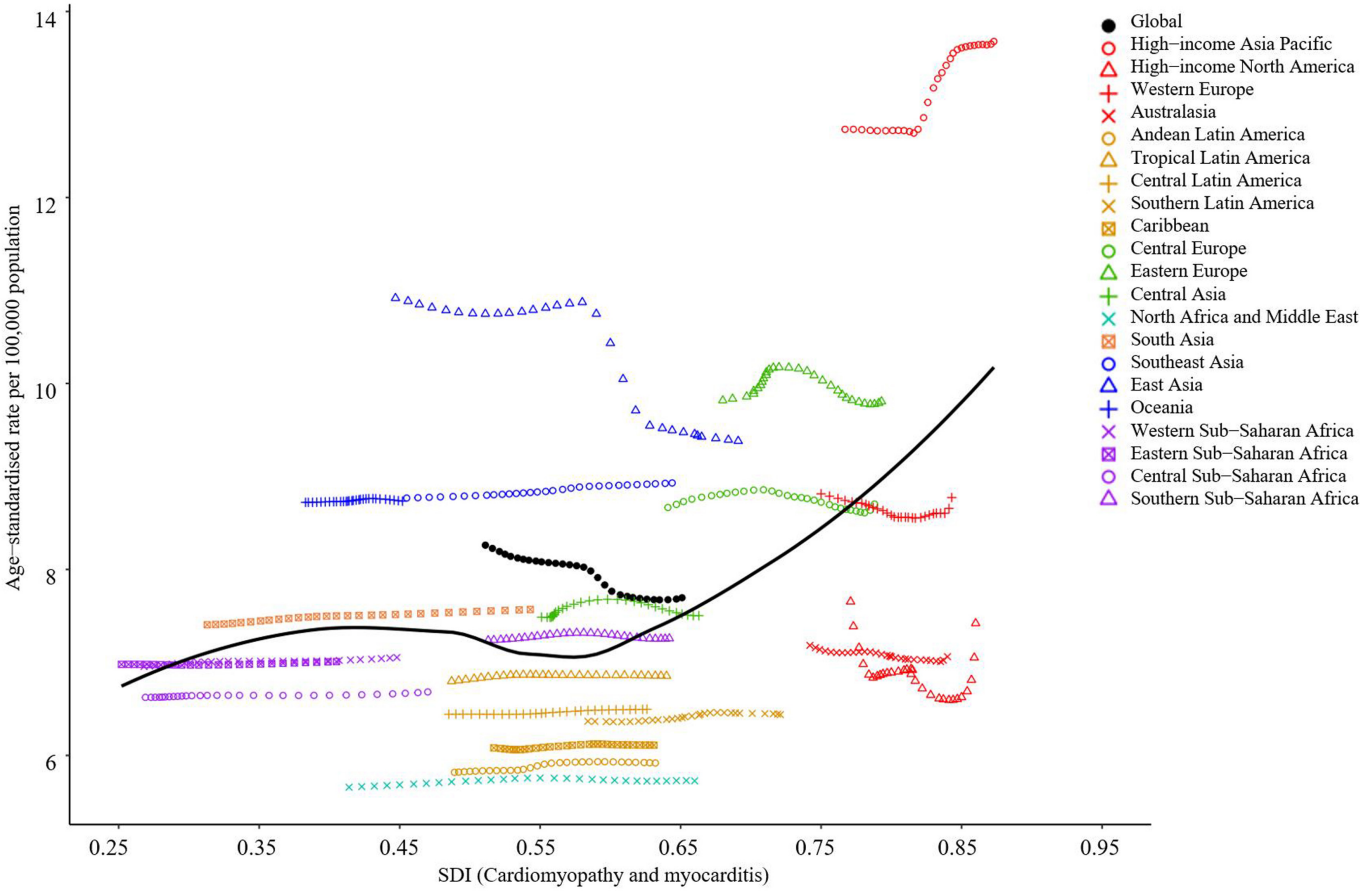
Age-standardized incidence of childhood myocarditis and cardiomyopathy per 100,000 children by 21 global regions and SDI, 2019. Globally, the age-standardized incidence of childhood myocarditis and cardiomyopathy declined with the increase in SDI. At the regional level, SDI changes in most areas did not make any meaningful difference in 2019. In East Asia, the increase of SDI was associated with a decrease of incidence rate. In High income Asia Pacific, the increase of SDI was associated with an increase of incidence rate. SDI, sociodemographic index.

At the regional level, SDI changes in most areas did not show meaningful differences in 2019 (Figure 2). In East Asia, the increase in SDI was associated with a decrease in incidence rate. In the high-income Asia Pacific, the increase in SDI was associated with an increase in incidence rate. Also, there were regions (Eastern Europe, Central Asia, and high-income North America) with more complex fluctuations without a clear trend although some patterns can be identified.

**Figure 3:**
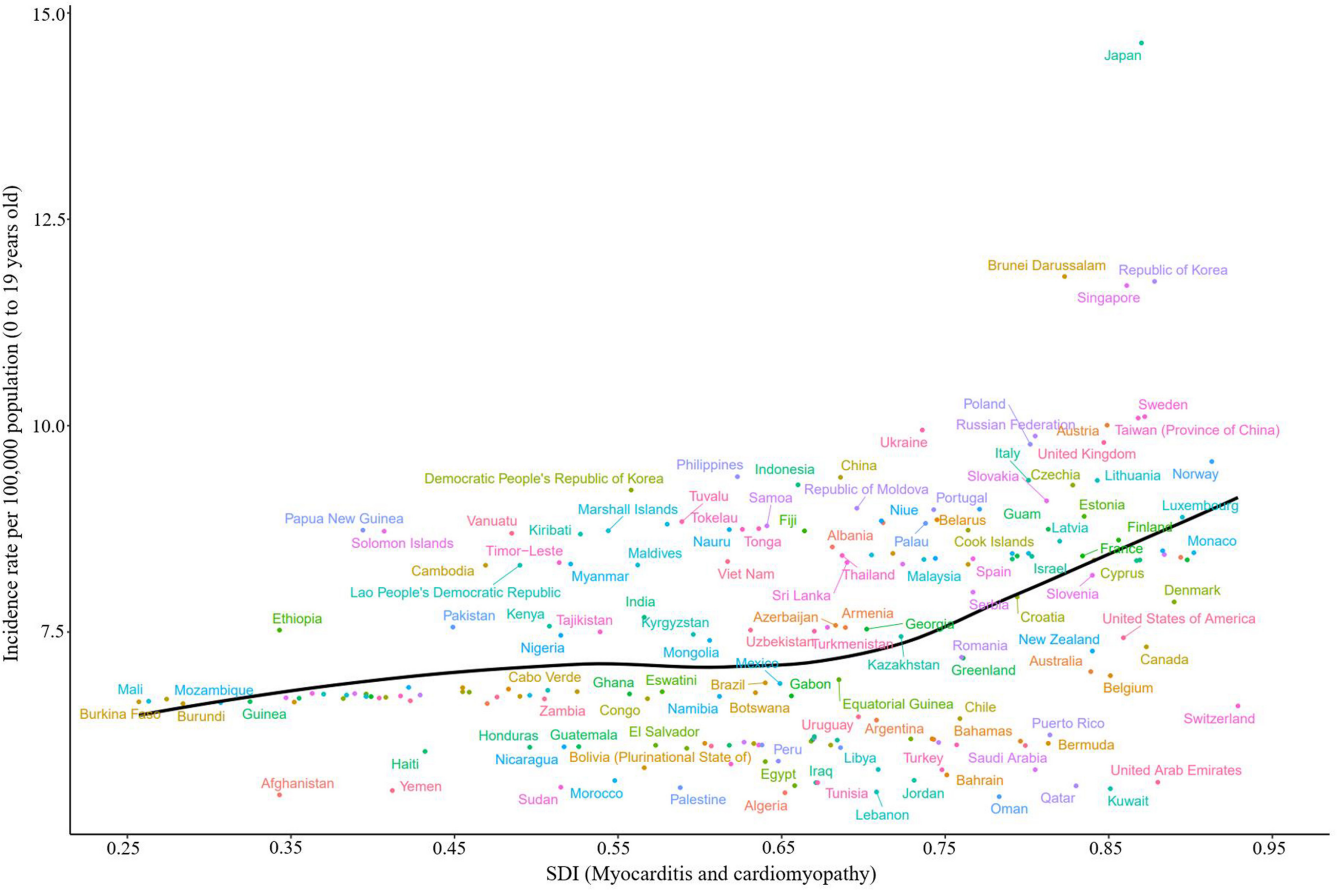
Age-standardized incidence due to childhood myocarditis and cardiomyopathy by country and SDI, 2019; the black line represents expected values. Globally, the age-standardized incidence of childhood myocarditis and cardiomyopathy increased with SDI in 2019. SDI, sociodemographic index.

**Figure 4:**
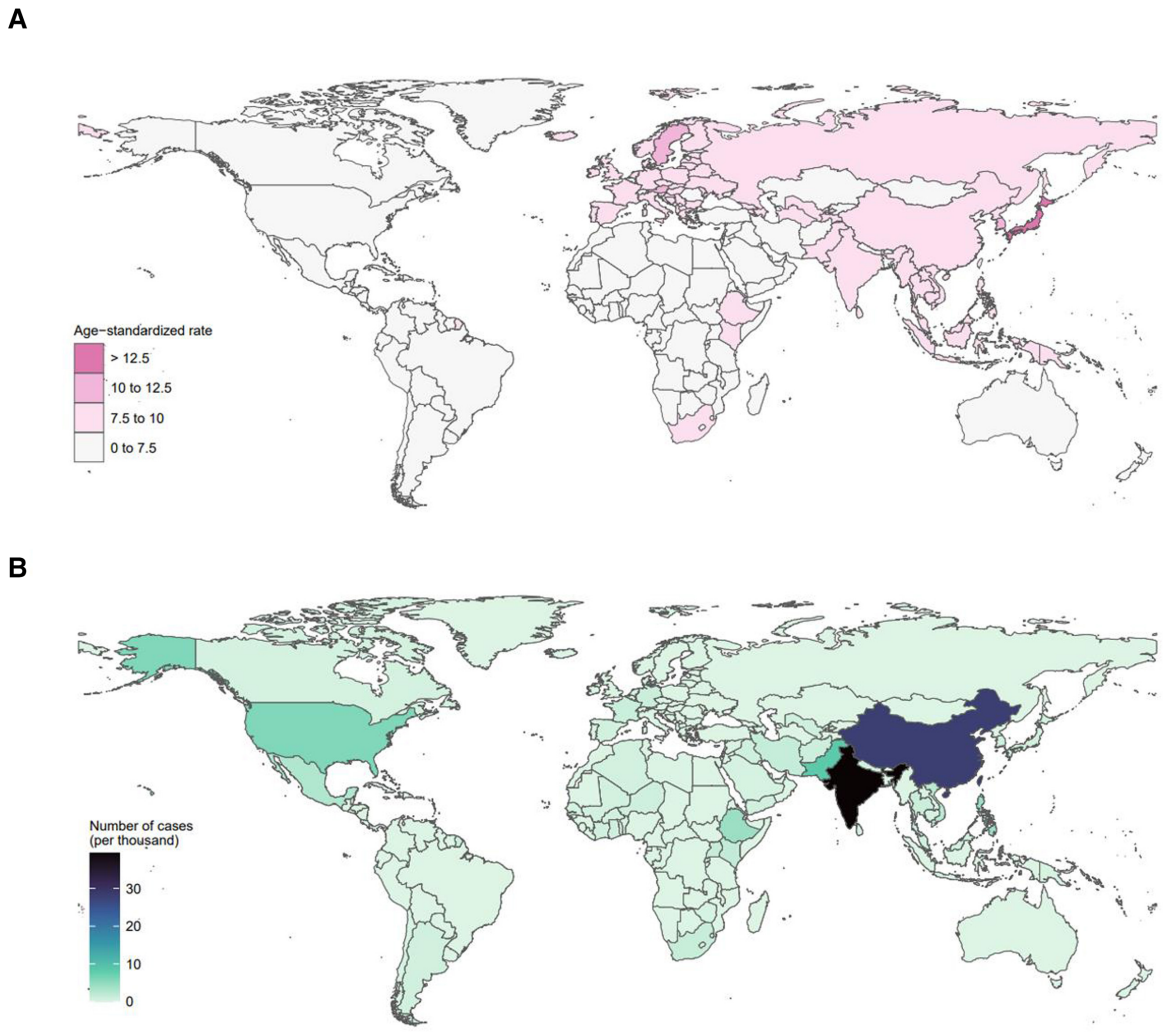
Global distribution map of childhood myocarditis and cardiomyopathy in 2019. (**A**) Global age-standardized incidence distribution map of childhood myocarditis and cardiomyopathy. In 2019, Japan had the highest age-standardized incidence of myocarditis and cardiomyopathy in children, followed by Sweden. (**B**) Global distribution map of the number of cases of myocarditis and cardiomyopathy in children in 2019. In 2019, India had the highest number of cases of myocarditis and cardiomyopathy in children, followed by China.

At the country level, the incidence of age-standardized childhood myocarditis and cardiomyopathy showed an increasing trend in 2019 as SDI increased, with Japan having the highest incidence and Afghanistan, the lowest (Figure 3 and Supplementary Table S2). Countries with the highest age-standardized incidence rates in 2019 were Japan and Sweden. However, the countries with the highest number of cases were India and China (Figure 4). Global incidence distribution map of childhood myocarditis and cardiomyopathy in different age groups in 2019 is shown in Supplementary Figure S1.

**Figure 5:**
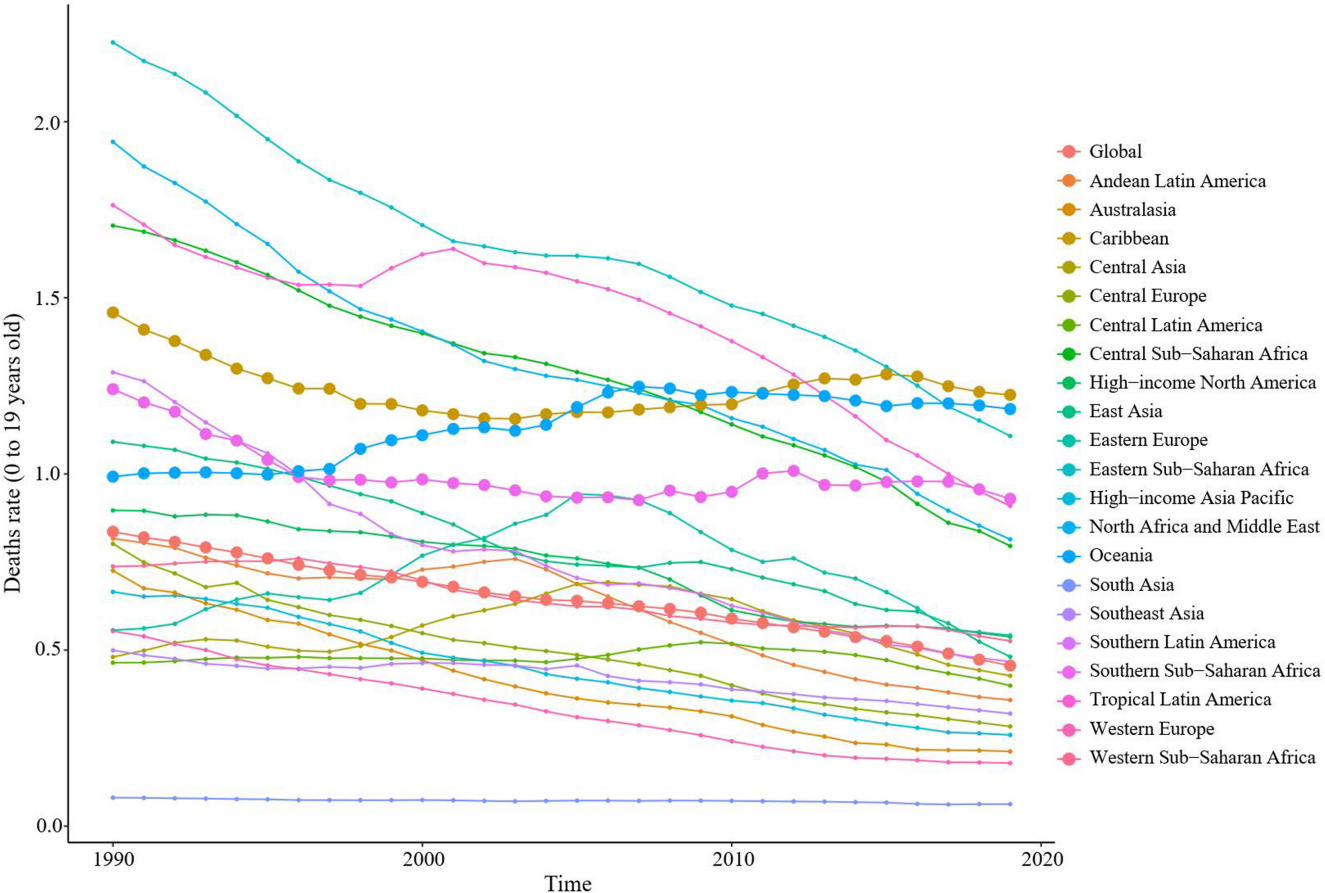
Trends in mortality of childhood myocarditis and cardiomyopathy in 21 regions from 1990 to 2019. From 1990 to 2019, the mortality rates from childhood myocarditis and cardiomyopathy showed a gradual decline in most regions of the world. However, it is on the rise in the Caribbean, Oceania and southern sub-Saharan Africa.

From 1990 to 2019, the mortality rates of childhood myocarditis and cardiomyopathy showed a gradual decline in most regions. However, it is on the rise in the Caribbean, Oceania and southern sub-Saharan Africa (Figure 5 and Supplementary Table S1). In 2019, 11,755 (95% UI 9,611–14,509) children died from myocarditis and cardiomyopathy worldwide. From 1990 to 2019, the age-standardized mortality rate decreased significantly by 0.4% (95% UI 0.2–0.6) to 0.5% (95% UI 0.4–0.6) (Tables 1, 2). In 2019, 6,775 (95% UI 5,287–8,781) boys died from childhood myocarditis and cardiomyopathy. The age-standardized mortality rate in boys in 2019 was 0.51% (95% UI 0.40–0.66) (Tables 1, 2). In 2019, 4,982 (95% UI 3,990–6,145) girls died from childhood myocarditis. The age-standardized mortality rate in girls was 0.40% (95% UI 0.32–0.49) in 2019 (Tables 1, 2).

**Table 2:** The burden of childhood myocarditis and cardiomyopathy by age group, sex and SDI in 1990 and 2019.

	Deaths (95% UI)		Incidence (95% UI)	
	1990 cases (thousands)	2019 cases (thousands)	1990 cases (thousands)	2019 cases (thousands)
**Age (years)**				
Under 5	14.17 (9.65–2.31)	7.44 (5.83–9.70)	47.00 (29.37–66.88)	45.63 (28.52–64.77)
5–9	1.43 (1.07–2.33)	1.16 (0.96–1.42)	44.38 (23.75–70.48)	45.63 (24.26–72.15)
10–14	1.32 (1.08–1.92)	1.20 (1.01–1.44)	44.87 (25.93–71.42)	49.82 (29.07–78.91)
15–19	2.08 (1.78–2.74)	1.96 (1.63–2.32)	51.56 (29.02–84.03)	57.40 (32.80–92.40)
**Sex**				
Male	10.17 (6.91–17.74)	6.78 (5.29–8.78)	114.50 (75.68–164.39)	121.26 (80.47–173.79)
Female	8.83 (6.17–14.37)	4.98 (3.99–6.15)	73.31 (48.25–105.83)	77.22 (50.68–111.54)
**Sociodemographic factor**				
Global	19.00 (13.71–30.12)	11.76 (9.61–14.51)	187.81 (123.59–270.18)	198.48 (130.82–285.81)
High SDI	1.68 (1.51–1.82)	0.77 (0.66–0.84)	21.32 (4.07–30.53)	19.36 (13.09–27.15)
High-middle SDI	3.37 (2.79–4.76)	1.36 (1.09–1.55)	36.08 (23.73–52.11)	27.22 (17.95–38.54)
Middle SDI	7.19 (5.04–13.49)	3.34 (2.69–4.28)	66.67 (44.15–95.88)	58.40 (38.40–84.06)
Low-middle SDI	3.13 (1.88–5.46)	2.22 (1.71–2.80)	43.22 (28.40–62.23)	51.77 (33.93–75.21)
Low SDI	3.63 (1.93–5.45)	4.07 (2.86–5.77)	20.52 (13.46–29.60)	41.73 (27.37–60.41)

UI, uncertainty interval; SDI, sociodemographic index.

### The burden in the under 5-year-old age group

**Figure 6:**
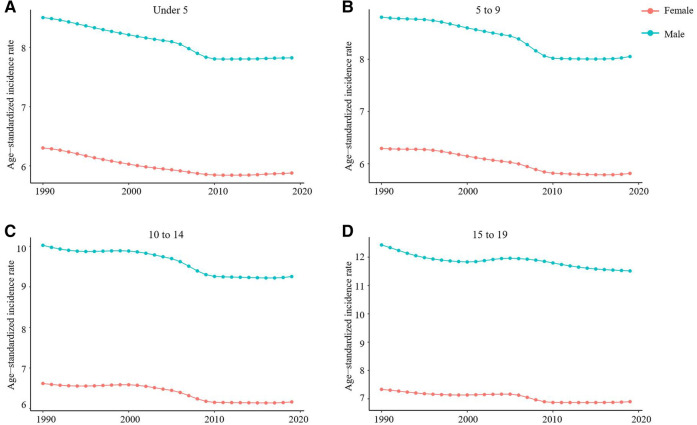
Trends in the age-standardized incidence of myocarditis and cardiomyopathy in children of different sex and age group, 1990–2019. (**A**) Change in trend of age-standardized incidence rate for under 5-year-olds. (**B**) Change in trend of age-standardized incidence rate for 5–9-year-olds. (**C**) The trend of age-standardized incidence in the 10–14 age group. (**D**). The trend of age-standardized incidence in the 15–19 age group.

**Figure 7:**
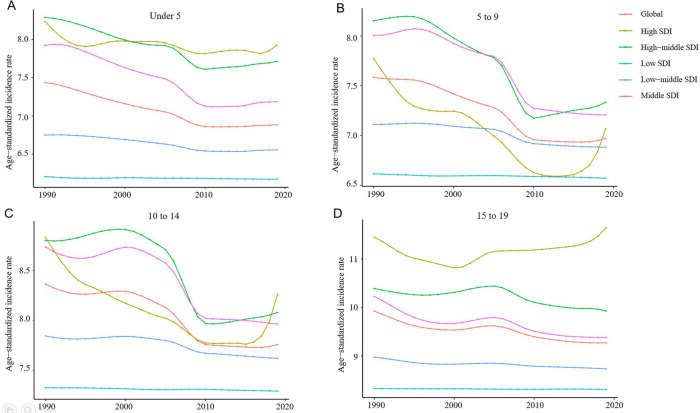
Change in trends of age-standardized incidence rate due to childhood myocarditis and cardiomyopathy by age group and SDI from 1990 to 2019. (**A**) Change in trend of age-standardized incidence rate for under 5-year-olds. (**B**) Change trends of age-standardized incidence rate for 5 to 9-year-olds. (**C**) Trends in age-standardized incidence rates in children aged 10 to 14 are similar to those of children aged 5–9. (**D**) Change in trend of age-standardized incidence rate for 15–19-year-olds. SDI, sociodemographic index.

**Figure 8:**
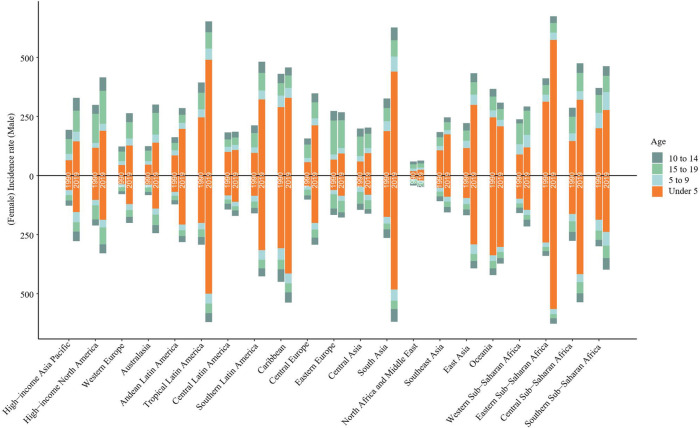
Changes in incidence rates due to childhood myocarditis and cardiomyopathy between 1990 and 2019 by age group in 21 global regions. The incidence of myocarditis and cardiomyopathy in children under five years of age increased significantly in 2019 compared to 1990 in all regions except Oceania. In both boys and girls, the incidence of myocarditis and cardiomyopathy increased in 2019 compared to 1990 in High−income Asia Pacific, Western Europe, and Australasia in children aged 15–19 years.

From 1990 to 2019, the incidence rate of myocarditis and cardiomyopathy in children under 5 years of age decreased globally (Figure 6A). Moreover, 45,631 (95% UI 28,518–64,766) children aged under 5 years appeared to have myocarditis and cardiomyopathy in 2019 (Table 2). The incidence rate of myocarditis and cardiomyopathy in children under 5 years of age decreased significantly in high-middle and middle SDI countries but decreased less in low-middle and low SDI countries (Figure 7). The incidence rate of myocarditis and cardiomyopathy in children under 5 years of age increased significantly in 2019 compared to 1990 in all regions except Oceania (Figure 8).

**Figure 9:**
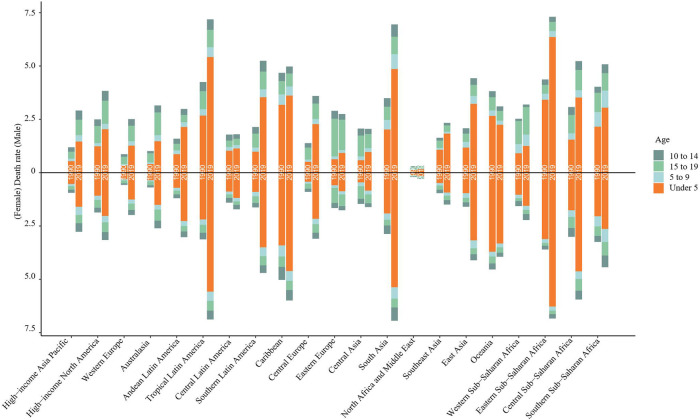
Changes in death rate due to childhood myocarditis and cardiomyopathy between 1990 and 2019 by age group in 21 global regions. The death rate of myocarditis and cardiomyopathy in children under five years of age increased significantly in 2019 compared to 1990 in all regions except Oceania.

**Figure 10:**
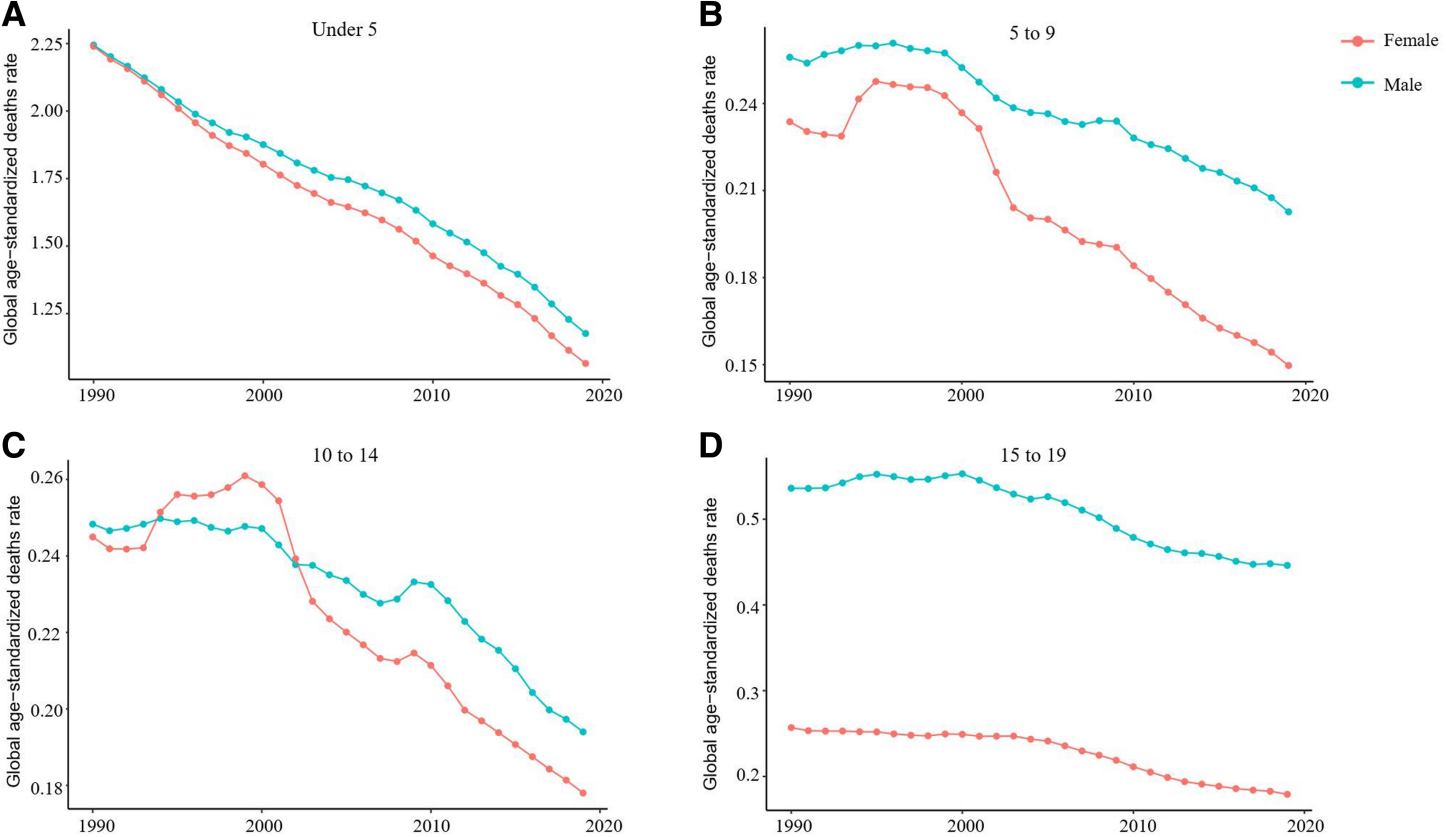
Change in trends of age-standardized death rate due to childhood myocarditis and cardiomyopathy by sex and age group from 1990 to 2019. (**A**) Change in trend of age-standardized death rate for under 5-year-olds. (**B**) Change in trend of age-standardized death rate for 5–9-year-olds. (**C**) Change in trend of age-standardized deaths rate for 10–14-year-olds. (**D**) Change in trend of age-standardized death rate for 15–19-year-olds.

**Figure 11:**
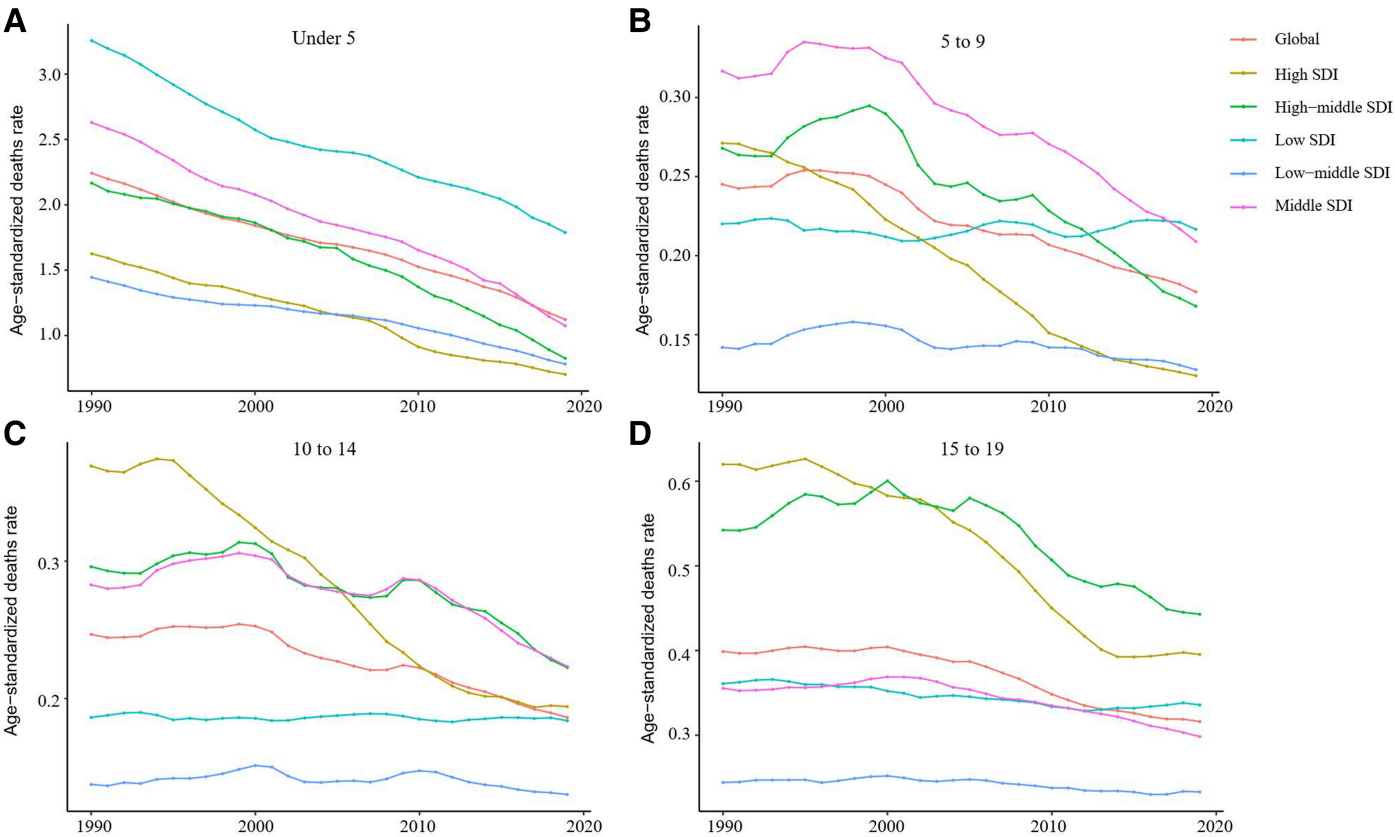
Change in trends of age-standardized death rate due to childhood myocarditis and cardiomyopathy by age group and SDI from 1990 to 2019. (**A**) Age-standardized death rates for myocarditis and cardiomyopathy in children under 5 years of age declined significantly from 1990 to 2019 in all regions with different SDI levels. (**B**) Change in trend of age-standardized death rate for 5–9-year-olds. (**C**) Change in trend of age-standardized death rate for 10–14-year-olds. (**D**) Change in trend of age-standardized death rate for 15–19-year-olds.

The number of deaths from childhood myocarditis and cardiomyopathy in 2019 was highest in the under 5-year-old group (7,442 (95% UI 5,834–9,699) (Table 2). From 1990 to 2019, the mortality rate in the under 5-year-old group decreased by 0.50% (95% UI 0.17–0.69) (Table 1).The death rate of myocarditis and cardiomyopathy in children under 5 years of age increased significantly in 2019 compared to 1990 in all regions except Oceania (Figure 9).The greatest reduction in myocarditis- and cardiomyopathy-related mortality was seen in girls (Figure 10A). From 1990 to 2019, the mortality rate of myocarditis and cardiomyopathy in children under 5 years of age decreased significantly in all SDI levels (Figure 11A).

### The burden in the 5- to 9 and 10- to 14-year-old age group

From 1990 to 2019, the incidence rate of myocarditis and cardiomyopathy in children aged 5–9 years declined significantly worldwide (Figure 6B); however, their rates in countries with low and low-middle SDI did not vary much (Figure 7B). The incidence of myocarditis and cardiomyopathy in children aged 5–9 years in middle SDI countries declined significantly (Figure 7B). However, it is noteworthy that the incidence rate of myocarditis and cardiomyopathy in children aged 5–9 years in high and high-middle SDI countries decreased significantly atfirst, and then increased (Figure 7B). The incidence rate in children aged 10–14 years (Figures 6C, 7C) is similar to that inchildren aged 5–9 years (Figures 6B, 7B).

The number of deaths due to childhood myocarditis and cardiomyopathy in the 5- to 9-year-old group in 2019 was 1,159, corresponding to an age standardized rate of 0.18%. The percent change in the mortality rate (from 1990 to 2019) decreased 0.28% (95% UI 0.02–0.52) (Tables 1, 2). The number of deaths due to childhood myocarditis and cardiomyopathy in the 10- to 14-year-old group in 2019 was 1,196, corresponding to an age standardized rate of 0.19%. The percent change in the mortality rate (from 1990 to 2019) decreased by 0.24% (95% UI 0.04–0.45) (Tables 1, 2). Change in trend of age-standardized death rate for 5–9-year-olds and 10–14-year-olds decreased significantly (Figures 10B,C).

From 1990 to 2019, the mortality rate of myocarditis and cardiomyopathy in children 5–9-year-olds and 10–14-year-olds decreased significantly in all SDI levels (Figures 11B,C).

### The burden in the 15- to 19-year-old age group

From 1990 to 2019, the incidence rate of myocarditis and cardiomyopathy among children and adolescents aged 15–19 years decreased globally (Figure 6D), and in middle and high-middle SDI countries (Figure 7D). In low-middle and low SDI countries, the incidence rates were stable. However, there was a clear upward trend in high SDI countries. In both boys and girls, the incidence of myocarditis and cardiomyopathy increased in 2019 compared to 1990 in high-income Asia Pacific, Western Europe, and Australasia in children aged 15–19 years (Figure 8). The number of deaths due to childhood myocarditis and cardiomyopathy in the 15–19-year-old group in 2019 was 1958, corresponding to an age standardized rate of 0.32%. The percent change in the mortality rate (from 1990 to 2019) decreased by 0.21% (95% UI 0.07–0.38) (Tables 1, 2). Change in trend of age-standardized death rate for 15–19-year-olds decreased significantly (Figure 10D). From 1990 to 2019, the mortality rate of myocarditis and cardiomyopathy in children 15–19-year-olds decreased significantly in all SDI levels (Figure 11D).

### Prediction of the incidence rate of myocarditis and cardiomyopathy in 2035

**Figure 12:**
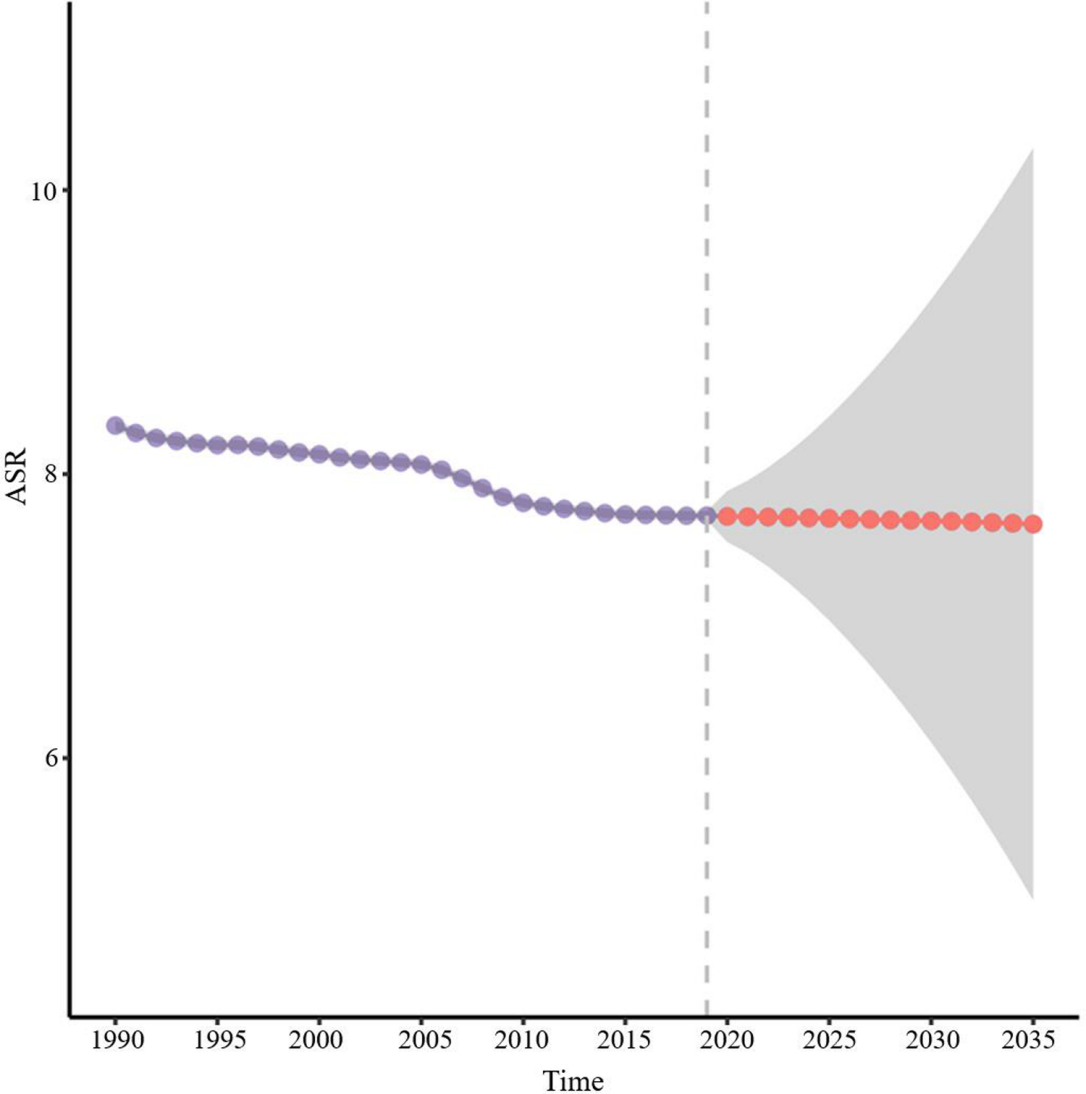
Prediction of the incidence rate of childhood myocarditis and cardiomyopathy for 2035. The age-specific incidence rate of childhood myocarditis and cardiomyopathy is projected to show a slow decline in 2035.

**Figure 13:**
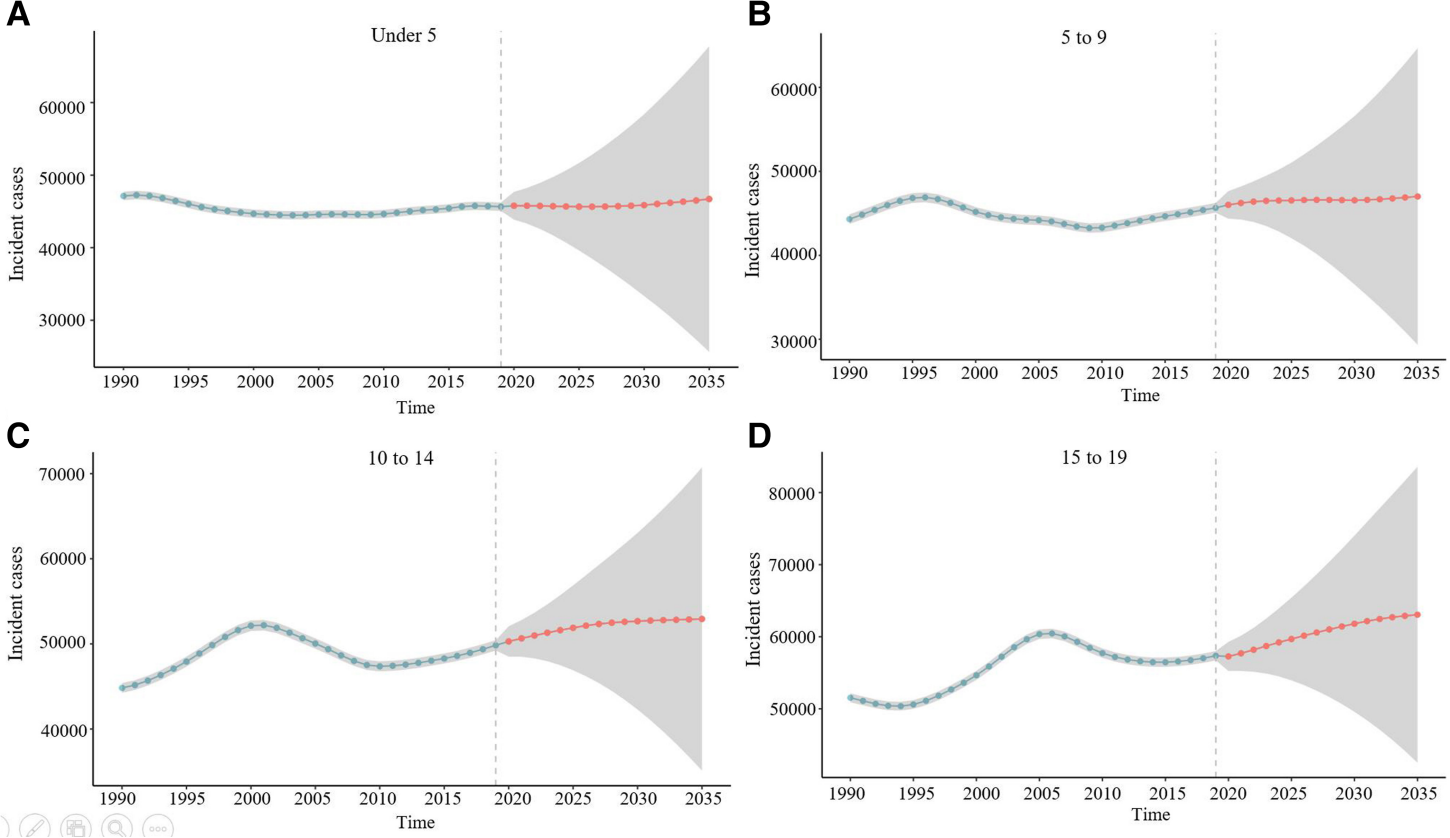
Prediction of the incidence rate of childhood myocarditis and cardiomyopathy by age group for 2035. (**A**) Prediction of the incidence rate of myocarditis and cardiomyopathy for under 5-year-olds. The number of cases of myocarditis and cardiomyopathy in 0–5 years old children is relatively stable, with the level at about 5,000 cases. (**B**) Prediction of the incidence rate of myocarditis and cardiomyopathy for 5–9-year-olds. There would be a slight increase in the incidence of myocarditis and cardiomyopathy in children aged 5–9 years. (**C**) Prediction of the incidence rate of myocarditis and cardiomyopathy for 10–14-year-olds. The incidence of myocarditis and cardiomyopathy in children aged 10–14 is projected to increase by 2035. (**D**) Prediction of the incidence rate of myocarditis and cardiomyopathy for 15–19-year-olds.

Based on the GBD study 2019, the age-standardized incidence rate of childhood myocarditis and cardiomyopathy is projected to show a slow decline in 2035 (Figure 12). The number of cases of myocarditis and cardiomyopathy in 0–5 year-old children was relatively stable, maintaining the level at about 50,000 cases (Figure 13). There was a slight increase in the incidence of myocarditis and cardiomyopathy in children aged 5–9 years. However, the incidence of myocarditis and cardiomyopathy in children aged 10–14 and 15–19 is projected to increase by 2035.

## Discussion

In this study, we used the GBD 2019 modelling framework to estimate the global burden of childhood myocarditis and cardiomyopathy. Stratified analysis was performed by age, sex, year, SDI, and region. To our knowledge, no previous studies on the burden of myocarditis and cardiomyopathy in children have been reported. In the present study, we found a steady decline inthe global incidence of childhood myocarditis and cardiomyopathy from 1990 to 2019. Furthermore, an interesting phenomenon occurred in the incidence of myocarditis and cardiomyopathy in children from countries where the SDI was positively proportional to the incidence. For example, the incidence of myocarditis and cardiomyopathy was relatively high in relatively developed countries, such as Japan, while it was relatively low in countries with low SDI, such as Afghanistan. The phenomenon has been found with other diseases as well (21, 22). Even though the exact cause is unclear, it is worth noting that the period studied in our paper, 1990–2019, was a period ofrapid advances in the understanding, diagnosis and management of cardiomyopathy and myocarditis (23–25). Effective myocarditis and cardiomyopathy screening strategies have been implemented in countries with high SDI (26–28). Similarly, in countries with high SDI, the use of cardiac magnetic resonance imaging allowed previously undiagnosed cases of myocarditis and cardiomyopathy to be identified (29, 30). In addition, from 1990 to 2019, the mortality due to childhood myocarditis and cardiomyopathy declined most significantly in high SDI countries. It also indirectly reflects the improved awareness of childhood myocarditis and cardiomyopathy in these areas, and the progress in diagnostic measures.

Childhood myocarditis and cardiomyopathy are one of the major causes of death in children (31). Our study shows that from 1990 to 2019, the global mortality rate of myocarditis and cardiomyopathy decreased in all age groups, with a significant decrease in children younger than 14 years of age, especially in those younger than 5 years. This may be attributed to the increasing research on myocarditis and cardiomyopathy in recent decades. The discovery of the role of immune mechanisms in the pathogenesis of myocarditis and cardiomyopathy (32, 33) and the widespread use of immunosuppressants may be the most important reasons (34, 35). In addition, countries with high SDI had the most significant reduction in mortality in patients of all ages. This further suggests that high SDI countries have better diagnosis and treatment strategies for childhood myocarditis and cardiomyopathy. In addition, in tropical Latin America, South Asia and eastern sub-Saharan Africa, myocarditis and cardiomyopathy deaths rate among children under 5 years of age increased significantly in 2019 compared with 1990. The exact reason for this is unclear. However, treatment strategies, such as the use of immunological agents, could have been based on the experience of countries with high SDI.

Another interesting finding of this study is that both the incidence and mortality rates of myocarditis and cardiomyopathy are lower in girls than in boys. Sex differences in the incidence of childhood myocarditis and cardiomyopathy have been reported in several studies (36, 37). There are several theories as to what could have caused this apparent sex difference. Some studies suggest that sex hormones have an important effect on several components of the immune system (38, 39). Epidemiological data suggest that oestrogen may have a protective effect while testosterone may promote myocarditis (40,41). Some authors also attribute these sex-related differences to men achieving higher levels of exercise and physical activity, especially in early adulthood (42). However, the specific mechanism needs to be further studied.

The age-standardized incidence of childhood myocarditis and cardiomyopathy from 1990 to 2019 is projected with a steady decline in the incidence in 2035. However, predictions by age group were inconsistent. The increasing incidence of myocarditis and cardiomyopathy in children over 10 years of age, especially in those aged 15–19 years, needs attention. The possible reasons for this trend are unclear and may be related to the increased incidence of childhood myocarditis and cardiomyopathy in high SDI regions (43). The specific reasons need to be further studied.

Although the GBD assessment fills a gap of scarce or inaccessible data on the disease burden of childhood myocarditis and cardiomyopathy, existing limitations should be fully recognized. First, data for some countries were incomplete, although statistical methods were used to overcome the data shortfalls and address the uncertainty. Data were missing and inaccurate in some countries, and of the results of the analyses relied on neighbouring countries' disease-related covariates and statistical trends. Besides, differences in data collection procedures and data quality in different countries, delays and inaccurate reporting, classification errors and coding biases are inevitable, although the GBD has made efforts to enhance the reliability and compatibility of relevant data. Finally, diagnostic criteria vary from time to time, which may reflect differences in the regional coding used.

In conclusion, global data on childhood myocarditis and cardiomyopathy from 1990 to 2019 show a decreasing trend in incidence and mortality, but an increasing trend in older children, especially in high SDI regions. Our analysis updates current world knowledge about the incidence and mortality of childhood myocarditis and cardiomyopathy. Our results encourage the continuation of current strategies in areas where childhood myocarditis and cardiomyopathy morbidity and mortality are well controlled. It also serves as a warning for those areas where the incidence and mortality are rebounding, to optimize prevention strategies and avoid further deterioration.

## Data Availability

The original contributions presented in the study are included in the article/**Supplementary Material**, further inquiries can be directed to the corresponding author.

## Author’s Note

Our study (Global burden of myocarditis and cardiomyopathy in children and prediction for 2035 based on the Global Burden of Patients Disease study 2019) and published papers (The Burden of Childhood Asthma by Age Group, 1990–2019: A Systematic Analysis of Global Burden of Disease 2019 Data) are not associated in any way, and the subjects are not the same.
