# Supplementary material for: Global burden of myocarditis and cardiomyopathy in children and prediction for 2035 based on the global burden of disease study 2019
Source: Front Cardiovasc Med. 2023 May 2;10:1173015. doi: 10.3389/fcvm.2023.1173015 (PMC10185772; doi:10.3389/fcvm.2023.1173015)
Supplement: Supplementary file 1 [file Datasheet1.pdf]

Map 0-5

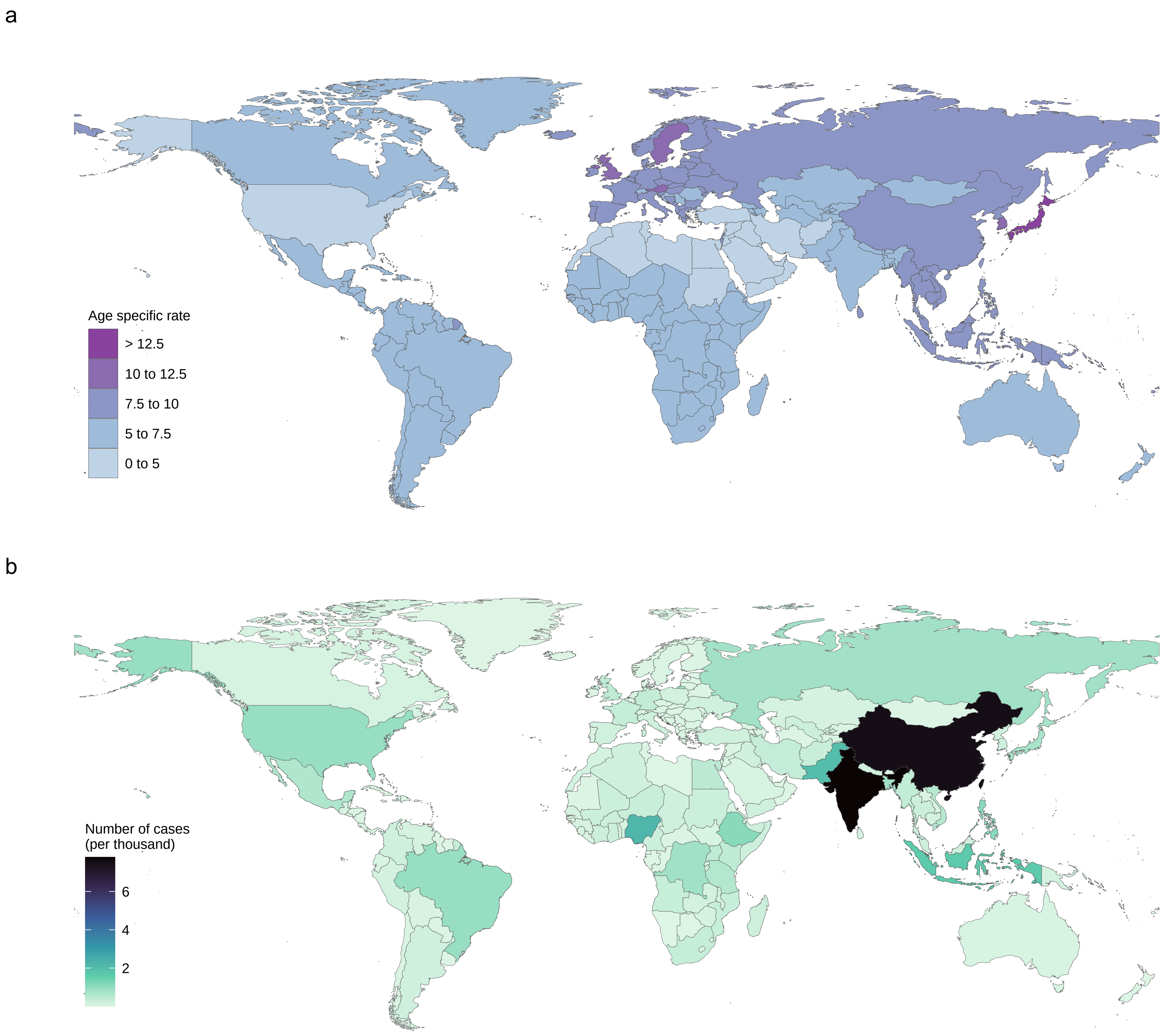

Figure S1A Global incidence distribution map of childhood myocarditis and cardiomyopathy in Under 5-Year-Old Age Group. a).Japan has the highest incidence. b).China and India have the most cases.

Map 5-9

a

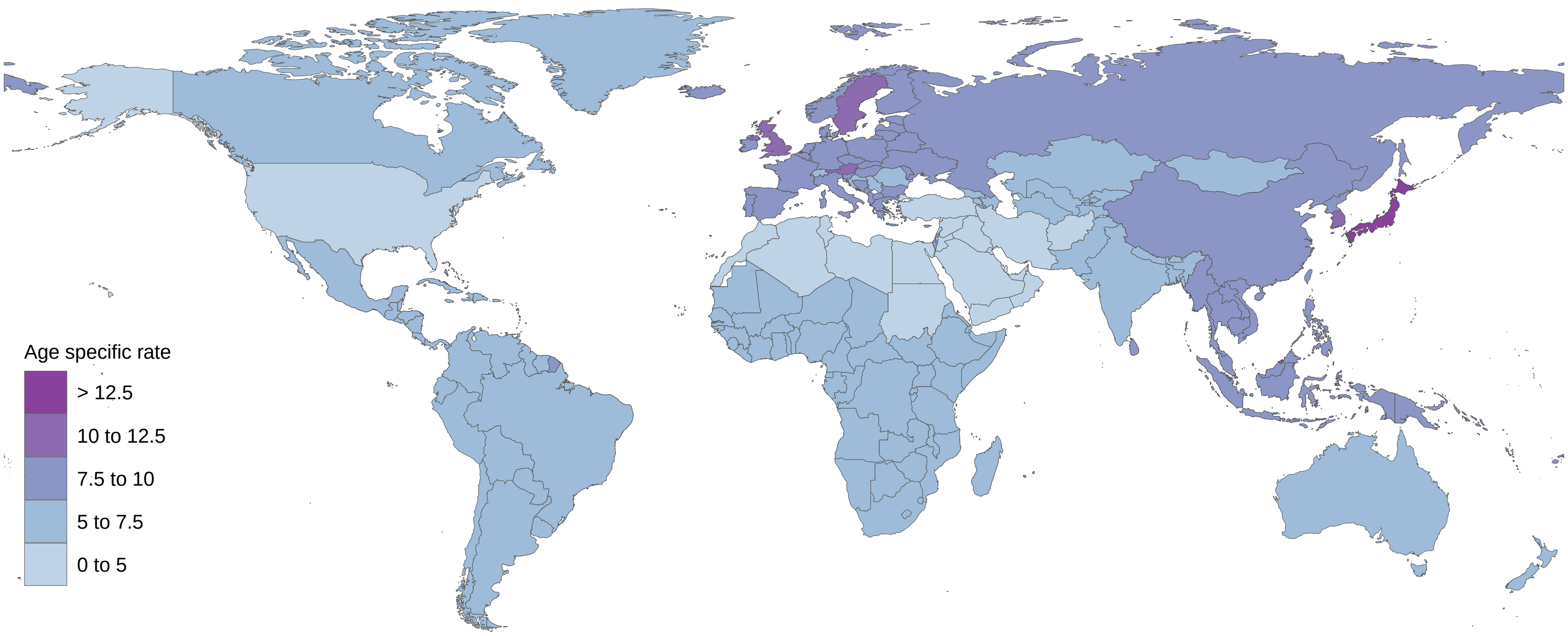

b

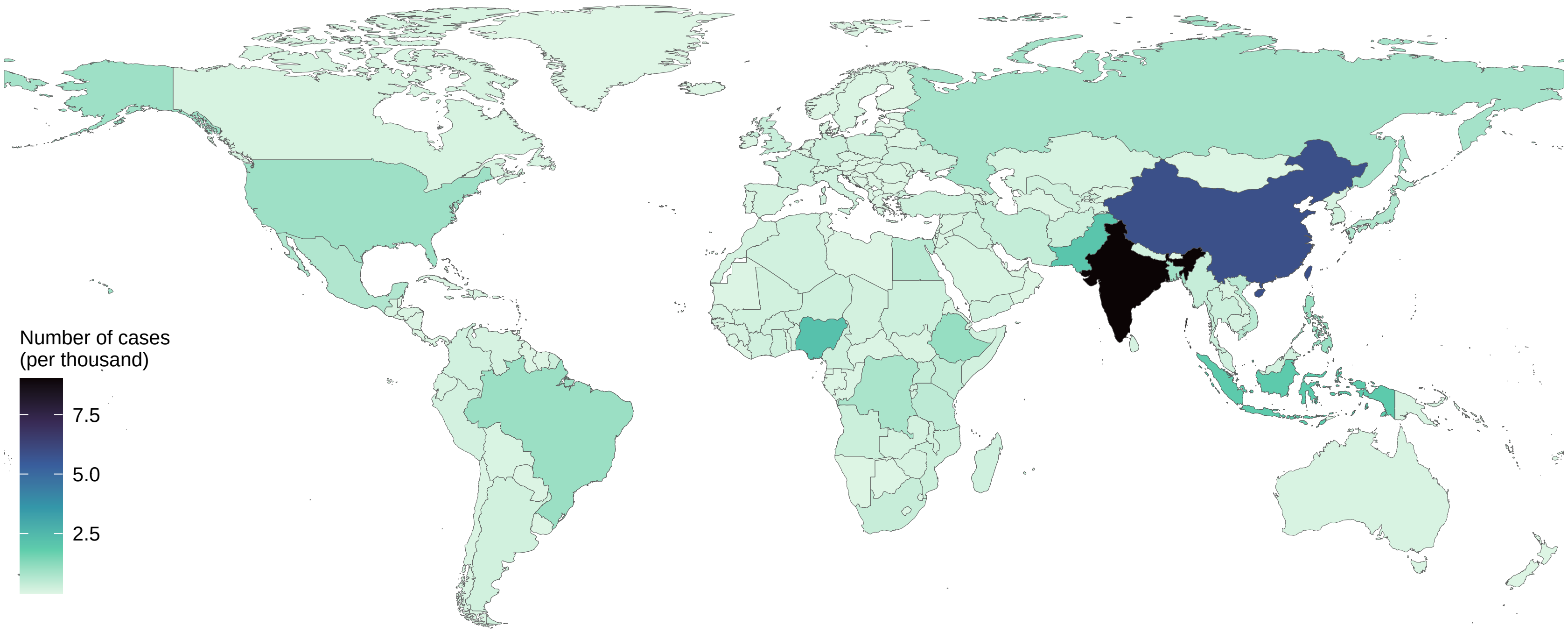

Figure S1B Global incidence distribution map of childhood myocarditis and cardiomyopathy in 5- to 9-Year-Old Age Group. a).Japan has the highest incidence. b).India have the most cases.

Map 10-14

a

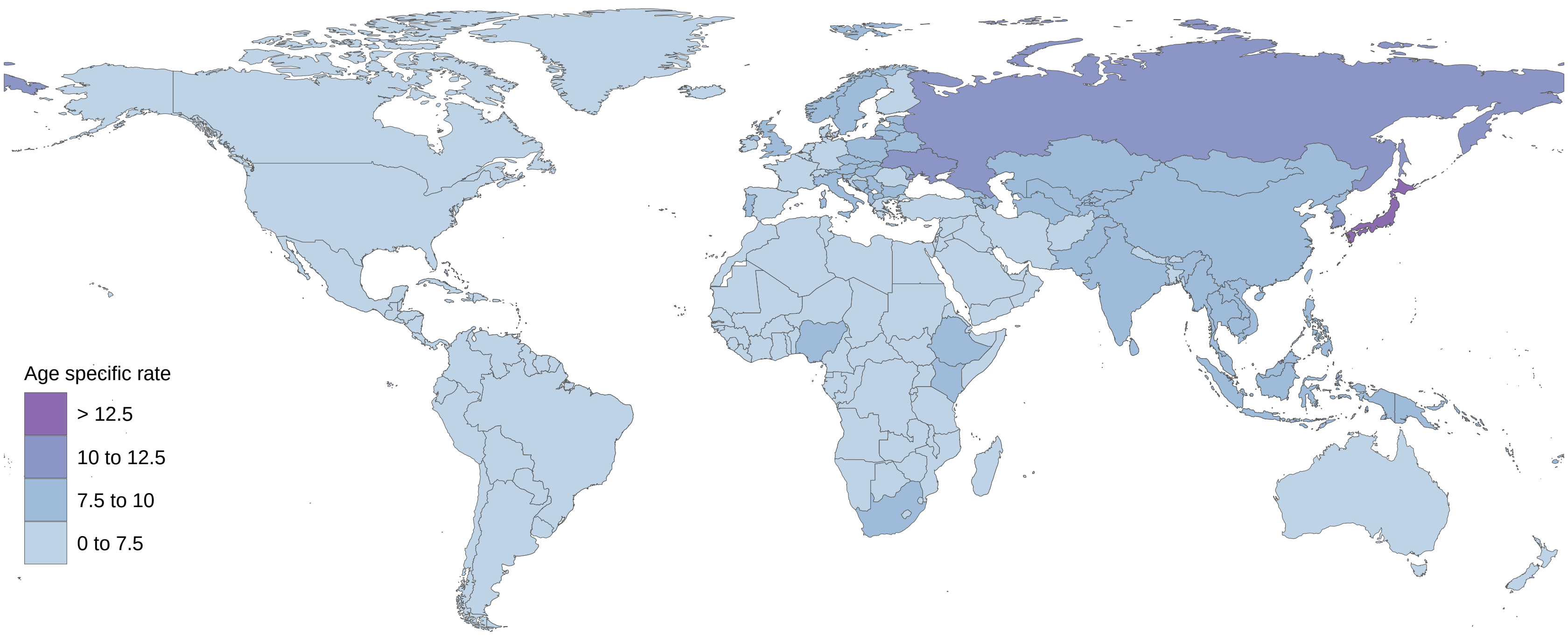

b

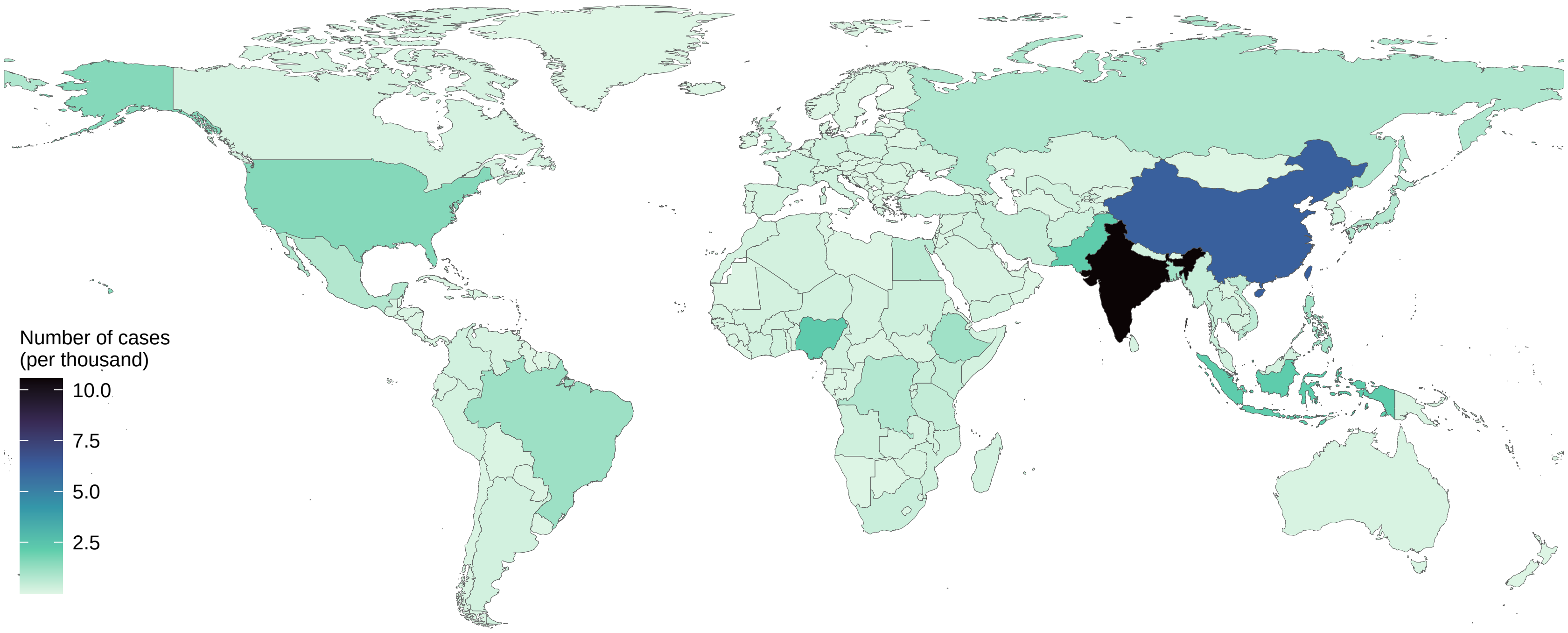

Figure S1C Global incidence distribution map of childhood myocarditis and cardiomyopathy in 10- to 14-Year-Old Age Group. a).Japan has the highest incidence.a).India have the most cases.

Map 15-19

a

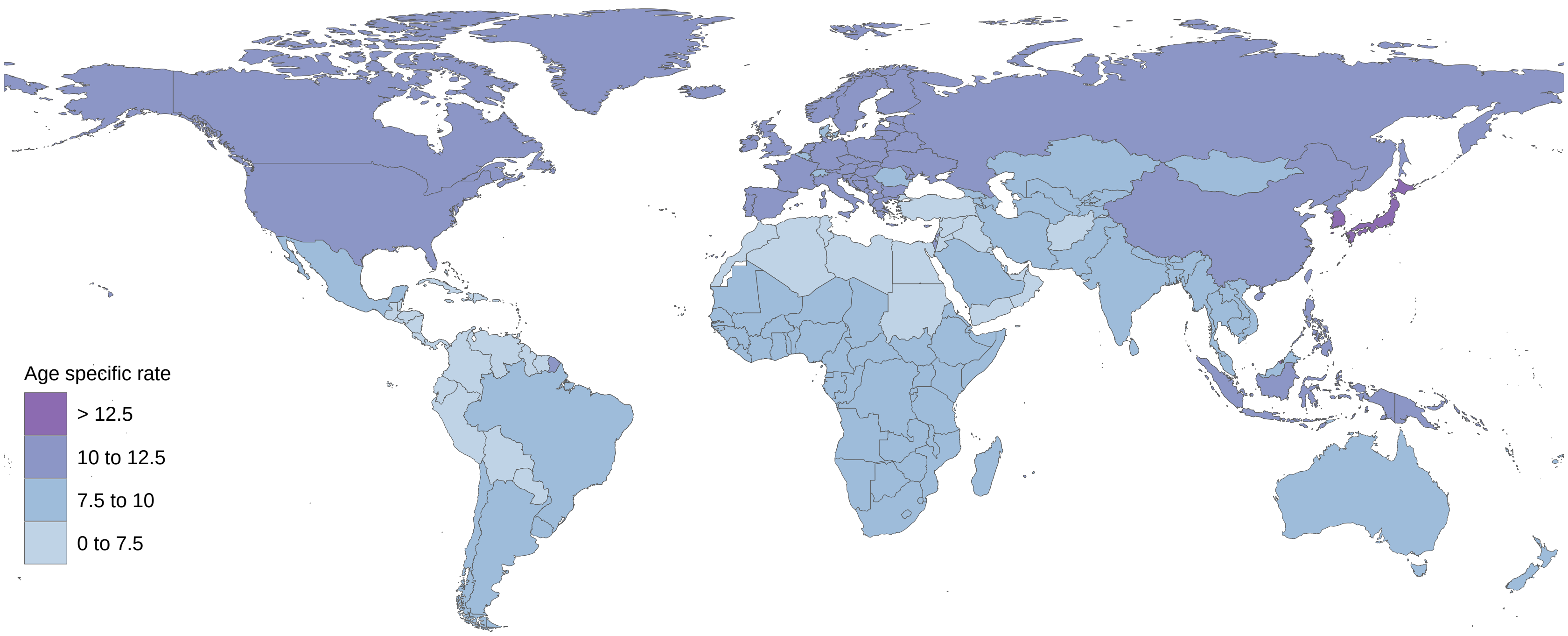

b

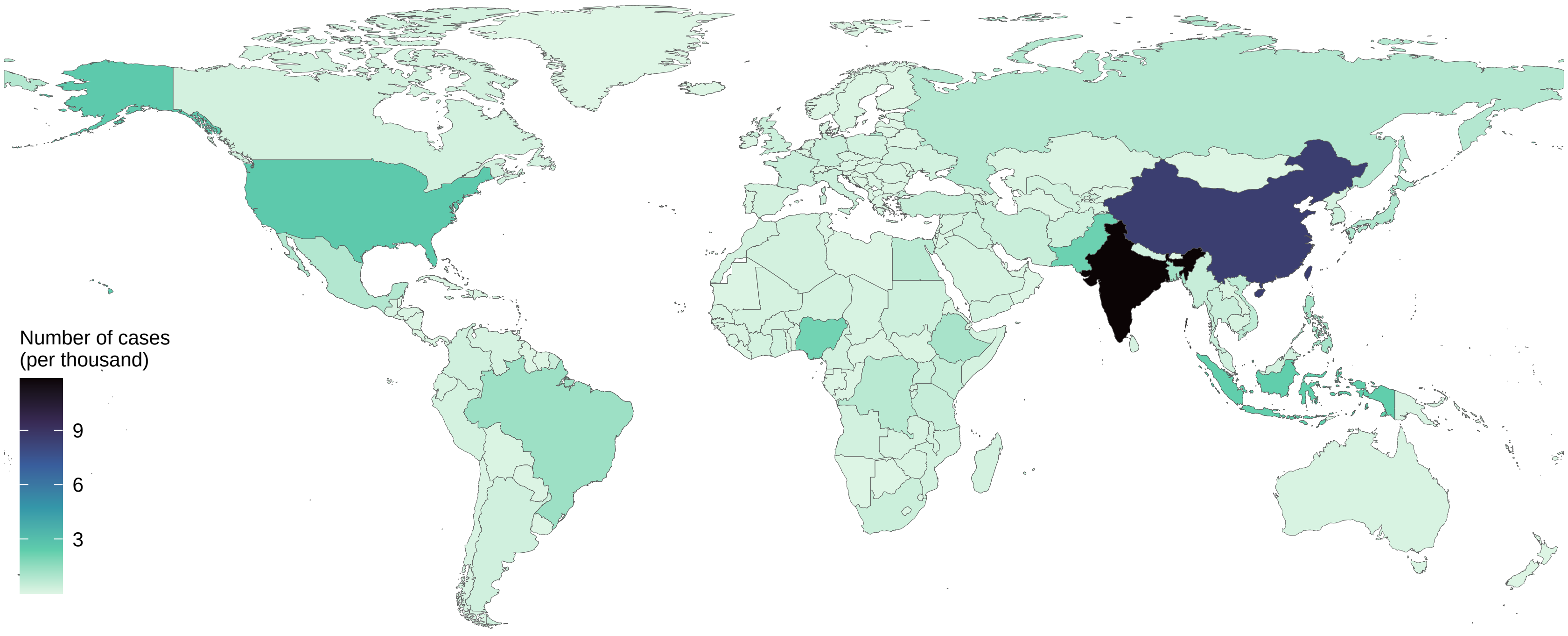

Figure S1D Global incidence distribution map of childhood myocarditis and cardiomyopathy in 15- to 19-Year-Old Age Group. a).Japan has the highest incidence. b).India and China have the most cases.

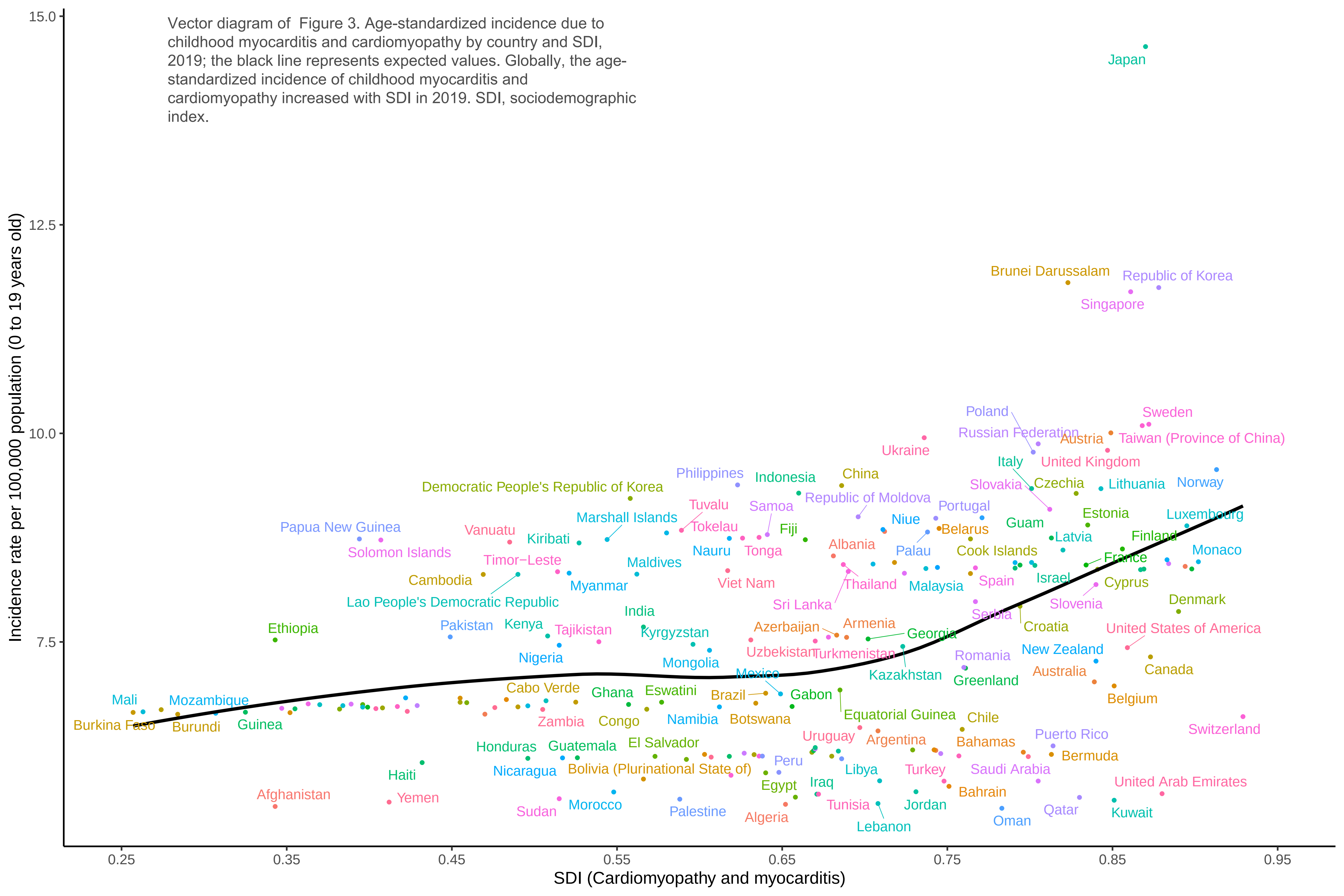

Vector diagram of Figure 3. Age-standardized incidence due to childhood myocarditis and cardiomyopathy by country and SDI, 2019; the black line represents expected values. Globally, the age-standardized incidence of childhood myocarditis and cardiomyopathy increased with SDI in 2019. SDI, sociodemographic index.
