# Supplementary figures and images for: Global burden of myocarditis and cardiomyopathy in children and prediction for 2035 based on the global burden of disease study 2019
Source: Front Cardiovasc Med. 2023 May 2;10:1173015. doi: 10.3389/fcvm.2023.1173015 (PMC10185772; doi:10.3389/fcvm.2023.1173015)

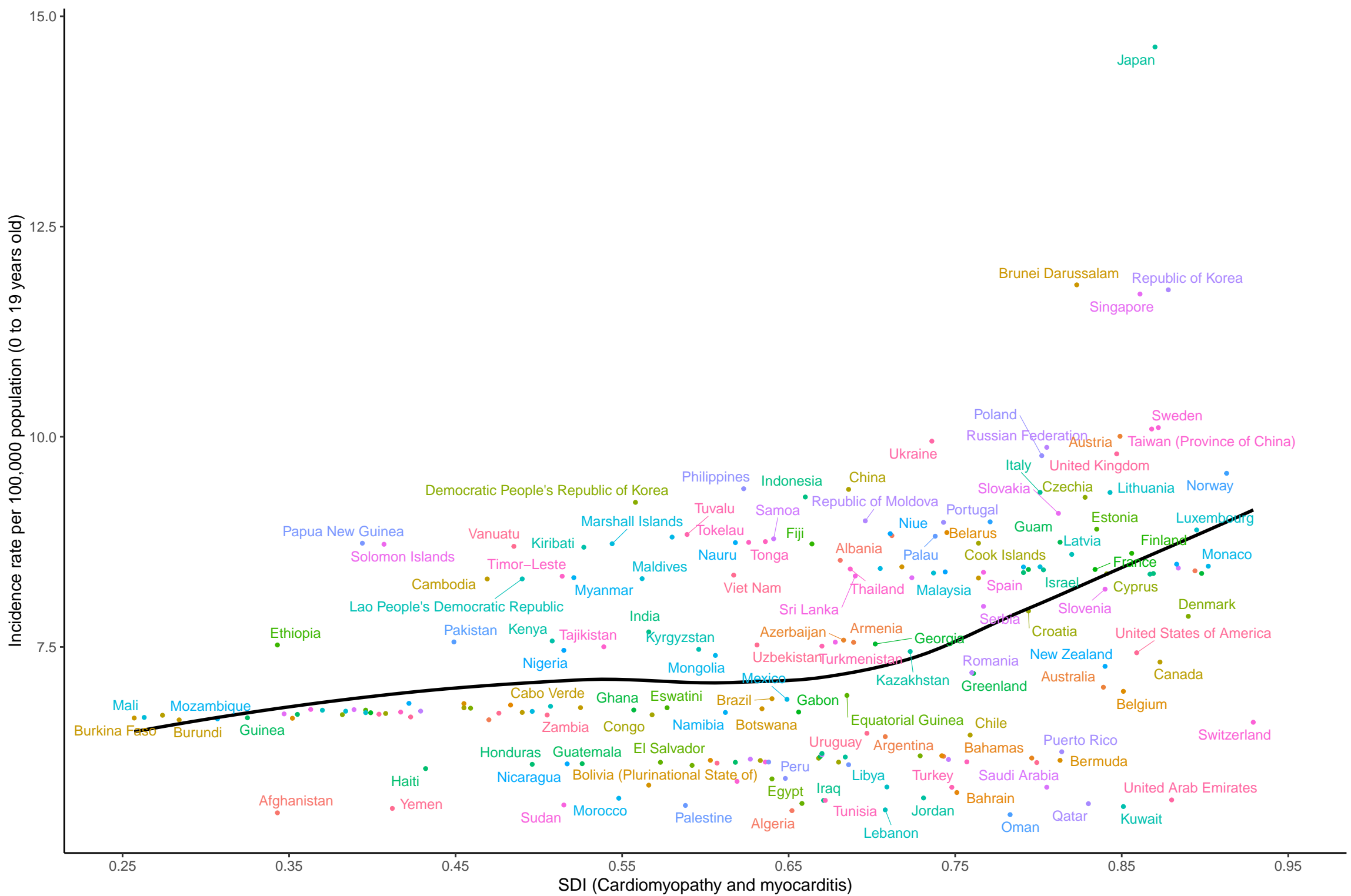

Supplement: Supplementary file 4 [file Datasheet4.pdf]
